# Supplementary material for: A scoping review of the impact of organisational factors on providers and related interventions in LMICs: Implications for respectful maternity care
Source: PLOS Glob Public Health. 2022 Oct 11;2(10):e0001134. doi: 10.1371/journal.pgph.0001134 (PMC10021694; doi:10.1371/journal.pgph.0001134)
Supplement: S2 Table — (DOCX) [file pgph.0001134.s002.docx]

# S2 Table

**Included non-intervention maternal health studies by region (N=41)**

| **Authors** | **Year** | **Country** | **Study Objective** | **Type of Study** | **Provider** | **Public/private/others** | **Level of facility** |
| --- | --- | --- | --- | --- | --- | --- | --- |
| Sub-Saharan Africa | | | | | | | |
| Prytherch et. al. | 2013 | Burkina Faso,  Ghana, Tanzania | Understand factors motivating maternal and neonatal health care providers; what influences their motivation, job satisfaction and quality of care | Qualitative | Multiple cadres (not specified) and managers at all levels | Public, private & faith-based | Primary |
| Pitchforth et. al. | 2010 | Ethiopia | Assess and understand issues affecting quality of clinical care in labour wards; identify priorities for change | Qualitative | Multiple cadres (not specified) | Public | Tertiary |
| Asefa et. al. | 2018 | Ethiopia | Understand service providers’ experiences of disrespect and abuse during facility-based childbirth | Qualitative | Midwives, nurses, health officers, medical doctors | Public | Multiple |
| Bogren et. al. | 2020 | DRC | Investigate midwives’  challenges and factors that motivate them to remain in their workplace; identify strategies that enable the optimal use of professional midwives | Quantitative | Midwives | Public & Private | Multiple |
| Adatara et. al. | 2021 | Ghana | Explore and gain insights into midwives’ experiences of working and providing women-centred care | Qualitative | Midwives | Public | Multiple |
| Dalinjong et. al. | 2018 | Ghana | Assess the availability of basic essential inputs including drugs, supplies and equipment in health facilities offering childbirth services; assess women and health provider’s perception of privacy and satisfaction with quality of care during childbirth | Mixed-methods | Midwives and nurses | Public | Multiple |
| Moyer et. al. | 2016 | Ghana | Determine what midwifery students witness, perceive, and learn with regard to respectful care during labour and childbirth | Quantitative | Midwifery students | Public midwifery schools | NA |
| Banchani and Tenkorang | 2014 | Ghana | Examine the challenges  confronting midwives in the implementation of maternal  health interventions and policies; to understand the challenges of maternal health logistics in health care facilities | Qualitative | Midwives and facility-level managers | Public | Tertiary |
| McKnight et. al. | 2020 | Kenya | Understand how neonatal nurses cope in resource constrained settings and how this affects practice | Qualitative | Nurses | Public | Secondary |
| Warren et. al. | 2017 | Kenya | To describe the types of mistreatment experienced by women during childbirth, and the perceived drivers of mistreatment | Qualitative | Policy makers, health managers, nurses, midwives, doctors & community based workers | A combination of public, private, faith-based | Multiple |
| Bradley et. al. | 2015 | Malawi | Explore the perceptions of EmOC providers on staff shortages and workload in their health facilities | Qualitative | Nurse-midwife technicians, nurses, midwives, clinical officers, medical assistants + managers | Public, Private & faith-based | Multiple |
| Chipeta et. al. | 2016 | Malawi | Understand how obstetric care staff perceive their working relationships with managers | Qualitative | Nurse-midwife technicians, nurses, midwives, clinical officers, medical assistants + managers | Public, Private & faith-based | Multiple |
| Mgawadere et. al. | 2019 | Malawi | Explore women's and healthcare provider's perspectives of what quality of care during childbirth means to them and how this can be improved | Qualitative | Medical officers, midwives, clinical officers and medical assistants | Public | Multiple |
| O’Donnell et. al. | 2014 | Malawi | Explore the perceptions of maternity care mothers and healthcare providers in a rural healthcare setting | Qualitative | Midwives, clinical officers, nurse attendant | Public | Tertiary + other |
| Maluwa et. al. | 2012 | Malawi | Assess knowledge and causes of moral distress among nurses, and coping mechanisms and sources of support used by nurses | Qualitative | Nurses | Public | Not specified |
| Pettersson et. al. | 2006 | Mozambique | Explore the midwives’ perception of factors obstructing or facilitating their ability to provide quality perinatal care | Qualitative | Midwives | Public | Tertiary |
| Wessen et. al. | 2018 | Namibia | Examine cultural  and structural factors that influence maternity care workers’ attitudes and practices; explore community perceptions about maternity care. | Mixed-methods | Mostly maternity care nurses, medical officers, matrons | Public | Tertiary |
| Ogu et. al. | 2017 | Nigeria | Explore women's perception of maternal health care providers’  workload and its effects on the delivery of maternal healthcare in secondary and tertiary hospitals | Qualitative | N/A | Public | Multiple |
| Matlala et. al. | 2019 | South Africa | Explore factors associated with the midwives’ intentions to stay or leave their primary employment in a public institution | Quantitative | Midwives | Public | Tertiary |
| Thopola & Lekhuleni | 2015 | South Africa | Explore and describe the challenges experienced  by midwifery practitioners in the midwifery practice environment | Qualitative | Midwives | Public | Primary |
| Bremnes et. al. | 2018 | Tanzania | Explore and highlight  the challenges midwives face in their day-to-day practice, and to investigate which measures the midwives find necessary to implement to improve their condition | Qualitative | Midwives | Public | Tertiary |
| Ng'ang'a et. al. | 2016 | Tanzania | Examine human resource management practices by comparing perspectives offered by mid-level  providers (MLPs) of emergency obstetric care (EmOC) in Tanzania to council health management teams (CHMTs) | Mixed-methods | Mid-level providers | Public | Multiple |
| Prytherch et. al. | 2012 | Tanzania | Explore motivation among Maternal and newborn healthcare (MNH) providers; factors encouraging and discouraging providers in rural areas; factors that influence providers’ performance and job satisfaction | Qualitative | Multiple cadres and managers at facility, district levels & above | Public and faith based | Multiple |
| Penfold et. al. | 2013 | Tanzania | Examine the experiences of professional staff providing maternal care in public rural health facilities in the context of poorly maintained equipment and insufficient drugs and supplies; to quantify the availability of functioning equipment and medical  supplies | l  Mixed-methods | Multiple cadres | Public | Multiple |
| Tibandebage et. al. | 2015 | Tanzania | Understand the role of managers in empowering nurse-midwives in resource poor hospitals | Qualitative | Nurse-midwives/maternity care | Public and faith-based hospital (publicly funded) | Tertiary |
| Sarkar et. al. | 2018 | Uganda | Understand how interpersonal dimensions of quality of care relate to real-life experiences of perinatal care, in a resource-constrained local  health system | Qualitative | Clinical officers, nurses, midwives | Public | Tertiary |
| Middle East & North Africa | | | | | | | |
| Rahmani & Bekele | 2013 | Afghanistan | Explore how pregnant women and health care providers experience antenatal and obstetric care. | Qualitative | Doctors, midwives and TBAs | Public, private & faith-based | Multiple |
| Hassan | 2017 | Egypt | Assess the implications of nurses' moral distress experience in clinical practice and their health status in maternal and  medical-surgical critical care settings | Quantitative | OBGYN, medical-surgical critical care nurses | Public | Tertiary |
| Samir et al. | 2012 | Egypt | Identify forms of workplace violence against obstetrics and gynaecology nurses and assess their reaction and attitude | Quantitative | OBGYN nurses | Public & Private | Tertiary |
| Shattnawi | 2017 | Jordan | Understand attitudes and behaviours of healthcare professionals toward breastfeeding practices and supporting mothers of preterm infants | Qualitative | nurses and physicians | Public | Tertiary |
| Hassan-Bitar | 2011 | Occupied Palestinian Territory | Explore the challenges and barriers faced by Palestinian maternal care providers to the provision of quality maternal health-care | Qualitative | midwives and doctors | Public | Tertiary |
| Abdulghani et. al. | 2020 | Saudi Arabia | Identify providers’ perceived facilitators, barriers and requirements for implementing the practice of Skin-to-Skin Contact (SSC) immediately after vaginal birth | Qualitative | obstetricians, midwives, and nurses. | Public | Tertiary |
| Turan et. al. | 2006 | Turkey | Document the current state of evidence-based obstetric practices. Identify provider attitudes, social pressures, and beliefs and patient preferences around non-evidence based practice | Mixed-methods | Maternity care providers; post-partum women | Public & private | Tertiary |
| Turan et. al. | 2006 | Turkey | Develop interventions to improve the quality of hospital-based maternity care | Mixed-methods | obgyn specialists, obgyn residents, midwives, nurses and aides | Public & private | Tertiary |
| Demirci et. al. | 2021 | Turkey | Understanding of the experiences and opinions of Turkish midwives  regarding the promotion of normal births | Qualitative | midwives | Public | Tertiary |
| Europe & Central Asia | | | | | | | |
| Fort and Voltero | 2004 | Armenia | Explore how factors,  individually as well as in combination, affect the performance of  nurse-midwives | Quantitative | Nurse-midwives | Public | Primary |
| Banovcinova et. al. | 2017 | Slovakia | Identify the most  common workplace stressors and most frequently used coping strategies among Slovak midwives, as well as the relationships between work place stressors, coping strategies, and demographic characteristics | Quantitative | midwives | Public |  |
|  | | | South Asia | | | | |
| Madhiwala et. al. | 2018 | India | Provide a better understanding of the institutional drivers of disrespect and abuse (D&A) in childbirth | Qualitative | doctors, residents, nurses | Public | Multiple |
| Mayra et. al. | 2021 | India | Investigate  the causes underlying mistreatment of women during childbirth by health care providers in India | Qualitative | midwifery and nursing leaders | N/A | N/A |
| Latin America & the Caribbean | | | | | | | |
| Miller et. al. | 2002 | Dominican Republic | Conduct a rapid assessment to understand a relatively high maternal mortality ratio despite nearly universal institutionalized deliveries with trained attendants | Qualitative | Nurses, general doctors, obstetrician, gynecologists, residents, interns, and students | Public | Multiple |
| North America | | | | | | | |
| Vania Smith-Oka | 2013 | Mexico | Explore the emergence of bureaucratic routinizing of obstetricians’ everyday practice | Qualitative and Quantitative | Clinicians (interns, residents, and full-time), nurses and midwives | Public | Tertiary |

# 
