# Supplementary material for: A scoping review of the impact of organisational factors on providers and related interventions in LMICs: Implications for respectful maternity care
Source: PLOS Glob Public Health. 2022 Oct 11;2(10):e0001134. doi: 10.1371/journal.pgph.0001134 (PMC10021694; doi:10.1371/journal.pgph.0001134)
Supplement: S3 Table — (DOCX) [file pgph.0001134.s003.docx]

# S3 Table

**Included intervention studies classified by type of response (N=13)**

| **Authors** | **Year** | **Country** | **Provider** | **Sector** | **Level of facility** | **Organizational factors** | **Departments/disciplines** | **Outcomes of interest** |
| --- | --- | --- | --- | --- | --- | --- | --- | --- |
| ***Planning for pressures and shortages*** | | | | | | | | |
| Ferdosi et. al. | 2013 | Iran | Nurses | Public | University hospital | Staff shortages | ENT and Neurosurgery | Total number of staff per bed and number of graduate nurses per bed increased. However, no notable increase in satisfaction with outsourced staff among administrators, managers, and supervisors. |
| Kaiser et. al. | 2019 | Zambia | Multi-cadre | N/A | Rural health centers | Infrastructure and time constraints | Maternity care | Added responsibilities for providers in the context of existing staff shortages. However, improved staff planning of their work, resulting in better, more timely care, thus feeling “better within their roles”. |
| Trap et. al. | 2001 | Zimbabwe | Pharmacy technicians and pharmacists |  | District level peripheral health facilities | Poor management of drugs | All departments | The pre–post effect of supervision on adherence to standard treatment guidelines among providers was found to be significant for all indicators, with an overall improvement of 22%. Improvement of clinical outcomes was observed in patients with infectious diseases. |
| ***Leadership focused change*** | | | | | | | | |
| Tuan et. al. | 2012 | Vietnam | Multi-cadre | Public | Tertiary | Leadership and organisational culture | CardiovascularRespiratory, GI, Endocrine, Neurology, Haematology, Paediatrics, Surgery, and Obstetrics | The study indicates that it improved professional relationships marked by improved knowledge-sharing and trust, providers being more patient and empathic in their care. |
| Jeon et. al. | 2018 | South Korea | Staff nurses | N/A | University hospital tertiary | Ethical leadership, organizational citizenship behaviour (OCB) | All departments | Improved overall scores for people orientation, task responsibility, relationship fairness, power sharing, concern for sustainability, ethical guidance and integrity. But pre and post-test differences were not statistically significant. |
| Mash et.al. | 2016 | South Africa | Facility manager, family physician, nursing managers, medical officer, pharmacist’s assistant, nurses in emergency centre and maternity unit, radiologist | Public | CHC | Organisational culture | Trauma unit, HIV unit, Maternity care | Significant (positive) change in the organizational culture. Cultural entropy (the level of dysfunction in an organization that is created as a result of limiting beliefs and fear-based behaviours of leaders) reduced from 33% to 13%. The culture moved away from hierarchy, control, and command, to -- communication, appreciation and accountability, among other positive changes. Also change in leadership styles enabling this transformation. |
| ***Providing Supportive supervision*** | | | | | | | | |
| Saadati et. al. | 2019 | Iran | Senior managers | Public | Tertiary | Patient safety | All departments | Changing the way patient safety is detected, reported and resolved was shown to improve teamwork and open communication between providers and management, and improved identification of patient safety incidents and their resolution. |
| Uduma et. al. | 2017 | Tanzania | Reproductive and child health personnel on the District Health Management Team and with obstetric care facility managers; health workers with supervisory responsibility and district level staff with supervisory responsibilities | Public | Multiple | Supportive supervision | All departments | Improvement in supervisory roles where a large majority of supervisors felt that they were much better at ‘treating staff with respect and recognizing their contribution’. Least improvement was seen regarding overall workload. Also helped remove some self-perceived barriers such as time management and lack of confidence. |
| Xie et. al. | 2017 | China | Nurses, nurse managers | Public | Multiple | Patient safety culture | All departments | Improved attitudes towards patient safety and perceptions of patient safety culture across dimensions such as open communication, non-punitive response to error, positive reinforcement for following standards, and teamwork. |
| ***Boosting resilience through peer support*** | | | | | | | | |
| Sook et. al. | 2016 | South Korea | Clinical nurses | N/A | General hospitals | Organisational commitment and empowerment | All departments | Measures of commitment and empowerment were significantly higher in the experimental group, but did not impact resilience significantly. The post-test, however, showed a lower turnover rate of nurses in the experimental group compared to the control group (4.2% vs. 20%). |
| ***Assessing and mitigating workplace violence*** | | | | | | | | |
| Coneo et. al. | 2020 | Uganda | Nurses and support staff | Public | Regional Referral Hospital | Workplace violence | Psychiatric care | Positive change of provider attitudes towards causes and management of patient aggression. Providers showed greater agreement with environmental and relational causative factors as main predecessors of aggression and were in favour of non‐physical methods. Although, attitudes towards: seclusion, medication and restraint remained unchanged. |
| Al- Ali et. al. | 2016 | Jordan | Nurses | Military hospital providing care for military and civilians | Tertiary care | Workplace violence | Critical care units (ICU, CCU, NICU, Burn unit), Medical-surgical  wards (Adult, Pediatric), Emergency department, and Outpatient clinics | Nurses post training showed more favourable attitudes towards the prevention of patient aggression to a more positive level, no significant changes found in the legal and safety standard. |
| Sharifi et. al. | 2020 | Iran | Nurses | N/A | University hospital | Workplace violence | Emergency department | Workplace violence reduced from a mean score of 8.4 to 2.7, which was statistically significant. There were significant differences in the frequency of verbal abuse, assessment of workplace security, fear of injury and type of reaction to violence before and after the intervention among the nurses. |
