# Supplementary material for: A scoping review of the impact of organisational factors on providers and related interventions in LMICs: Implications for respectful maternity care
Source: PLOS Glob Public Health. 2022 Oct 11;2(10):e0001134. doi: 10.1371/journal.pgph.0001134 (PMC10021694; doi:10.1371/journal.pgph.0001134)
Supplement: S1 Text — (DOCX) [file pgph.0001134.s004.docx]

# S1 Text

PubMed search strategy

| Org conditions (broad) | "organizational factor*"[All Fields] OR "organizational attribute*"[All Fields] OR "organizational environment"[All Fields] OR "working conditions"[All Fields] OR "working environment"[All Fields] OR "workplace culture"[All Fields] OR "organizational climate"[All Fields] OR "professional culture"[All Fields] OR "organizational culture"[tw] OR "organization and administration"[Majr] |
| --- | --- |
| Org conditions (specific) | "organizational support" OR "Decision Making", Organizational"[Mesh] OR "physical infrastructure" OR "Change Management"[Mesh] OR "Workflow"[Mesh] OR "Personnel Staffing and Scheduling"[Mesh] OR "Crew Resource Management, Healthcare"[Mesh] OR "Professional Autonomy"[Mesh] OR "Leadership"[Mesh] OR "Workload"[Mesh] OR "task shifting" OR "resource constraints" OR "staff shortages OR "Health Care Rationing"[Mesh] OR "occupational stress" [MeSH] OR "workplace violence" |
| Care outcomes | "Quality Assurance, Health Care"[Mesh] OR "Culturally Competent Care"[Mesh] OR "Patient Safety"[Mesh] OR "Patient compliance" [Mesh] OR "Guideline Adherence"[Mesh] OR "Patient Comfort"[Mesh] OR "Empathy"[Mesh] OR "Ethics, Medical"[Mesh] OR "Standard of Care"[Mesh] OR "Attitude of Health Personnel"[Mesh] OR "Professional-Patient Relations"[Mesh] OR "respectful maternity care" OR "respectful maternal care" OR disrespect* OR abus* OR mistreatment OR neglect OR "Patient Rights"[Mesh] OR Patient Satisfaction[Mesh] OR "client satisfaction" OR "Patient-Centered Care"[Mesh] OR "person-centered maternity care" OR "obstetric violence" |

Scopus search strategy

| Org conditions (broad) | "organizational factor*"  OR  "organizational attribute*"  OR  "organizational environment"  OR  "working conditions"  OR  "working environment"  OR  "workplace culture"  OR  "organizational climate"  OR  "professional culture"  OR  "organizational culture" |
| --- | --- |
| Org conditions (specific) | support  OR  "Decision Making"  OR  "physical infrastructure"  OR  workflow  OR  "Professional Autonomy"  OR  leadership  OR  workload  OR  "task shifting"  OR  "resource constraints"  OR  "staff shortages"  OR  "Health Care Rationing"  OR  "occupational stress"  OR  "workplace violence" |
| Care outcomes | "quality of care"  OR  "Culturally Competent Care"  OR  "Patient Safety"  OR  "Patient compliance"  OR  "Guideline Adherence"  OR  "Patient Comfort"  OR  empathy  OR  ethics  OR  attitude  OR  behaviour  OR  "provider-patient"  OR  "patient-provider"  OR  "respectful maternity care"  OR  disrespect*  OR  abus*  OR  mistreatment  OR  neglect  OR  "Patient Rights"  OR  "Patient Satisfaction"  OR  "client satisfaction"  OR  "Patient-Centered Care"  OR  "obstetric violence" |
