# Supplementary material for: A scoping review of the impact of organisational factors on providers and related interventions in LMICs: Implications for respectful maternity care
Source: PLOS Glob Public Health. 2022 Oct 11;2(10):e0001134. doi: 10.1371/journal.pgph.0001134 (PMC10021694; doi:10.1371/journal.pgph.0001134)
Supplement: Attachment — Submitted filename: Response to Reviewers.pdf [file pgph.0001134.s005.pdf]

Dear Dr. Tappis,

We are submitting the revised manuscript, “A scoping review of the impact of organisational factors on providers and related interventions in LMICs: Implications for Respectful Maternity Care”. We thank the reviewers for their careful consideration and very useful feedback. Please see the table below for responses to each comment. Line numbers in the responses refer to the document ‘Revised Manuscript with Track Changes’.

Sincerely,

Bhavya Reddy (on behalf of all authors)

Ramalingaswami Centre on Equity and Social Determinants of Health
Public Health Foundation of India

Editor comments	Author responses
1. Please amend your detailed online Financial Disclosure statement. This is published with the article. It must therefore be completed in full sentences and contain the exact wording you wish to be published.<br><br>State what role the funders took in the study. If the funders had no role in your study, please state: “The funders had no role in study design, data collection and analysis, decision to publish, or preparation of the manuscript.”	The financial disclosure statement now reads “ This work was funded by UNDP/UNFPA/UNICEF/WHO/World Bank Special Programme of Research, Development and Research Training in Human Reproduction (HRP), a cosponsored program executed by the World Health Organization (WHO). The funders had no role in study design, data collection and analysis, decision to publish, or preparation of the manuscript.”
4. Please update your online Competing Interests statement. If you have no competing interests to declare, please state: “The authors have declared that no competing interests exist.”	The wording of the Competing Interests statement has been changed to reflect this.
4. Please provide a complete Data Availability Statement in the submission form. If your research concerns only data provided within your submission, please write “All data are in the manuscript and supporting information files.” As your Data Availability Statement	Yes, therefore the wording of the Data Availability statement has been modified to reflect this.
4. Please provide separate figure files in .tif or .eps format and remove any figures embedded in your manuscript file. Please also ensure that all files are under our size limit of 10MB.	All figures have been removed from the manuscript and uploaded as .tif files and are under 10MB.
5. Please ensure that you refer to your figures in your text as, if accepted, production will need these references to link the reader to the figures.	The recommended language has been used to refer to figures in the manuscript and appear in track changes.

6. We noticed that you have two Figure 1's in your manuscript. Please update your figure numbers and cite them accordingly.	These have been changed to Fig 1, Fig 2 and so forth.
7. Tables cannot contain images. Please remake any tables with images as main figures and provide them as separate one page .tif or .eps files. Please change any in-text citations as necessary.	The table image has been removed and attached as .tif file and referred to as recommended.
8. We have noticed that you have uploaded Supporting Information files, but you have not included a list of legends. Please add a full list of legends for your Supporting Information files after the references list.	A list of legends has been provided after the references list and appear in track changes.
9. Please review your reference list to ensure that it is complete and correct. If you have cited papers that have been retracted, please include the rationale for doing so in the manuscript text, or remove these references and replace them with relevant current references. Any changes to the reference list should be mentioned in the rebuttal letter that accompanies your revised manuscript. If you need to cite a retracted article, indicate the article's retracted status in the References list and also include a citation and full reference for the retraction notice.	To our knowledge, the reference list contains no retracted papers. References that have been added appear in track changes.
**Reviewer 1<br>Major comments followed by related annotated comments**	**Author responses**
4) The choice to dichotomize by country income levels should be examined and substantiated. There is arguably more generalizability across the range of country income levels with respect to maternal health medical culture, organizational issues, and RMC, than there is across health disciplines. D&A is known to be a universal phenomenon whose manifestations and their magnitude may vary by context. As a form of gender-based violence the link to women's sexual and reproductive health is not incidental. The rationale and support for the extension of the search criteria to include studies from other medical disciplines should be explicitly shared.<br><br>Is D&A the same for all other disciplines?<br><br>I would argue that there would have been more parallels and generalizability between social and organizational issues in maternal health systems across country income levels than there is across medical disciplines.<br><br>Maternal health is a specific culture, that is part of women's productive health, and as such is	Our purpose for this review was to understand facility-level organisational factors that drive poor provider behaviour and care. While recognising that gender adverse behaviours and practices in health facilities are heightened in the Obstetrics and Gynaecology departments, our reasons for drawing lessons for RMC from across medical departments rather than from HIC maternity care are as follows.<br><br>**Severity of shortages:** The literature drew our attention to how resource shortages impact providers in both HICs and LMICs. However, we believe the magnitude and implications of shortages in HICs is nowhere near what is evidenced in LMICs. The shortage of nurses, for example is a global problem, but much more acute in LMIC contexts (reflected in examples like a single nurse being responsible for an entire ward or two wards in papers reviewed). Many analyses of poor care in LMICs begin and end with resource shortages. But we were interested in how the effects of shortages can be buffered or exacerbated by other organisational factors.<br><br>**Health system context/structure:** Facility level factors are shaped by how health systems are organised and financed. In this respect, LMICs

subject to specific and distinct ideologies and power hierarchies that do not compare with other medical domains where gender and sexual and reproductive health are not at issue.	differ hugely from HICs. The majority of our included studies were set in public facilities in LMICs with common funding challenges and inefficiencies, such as delayed salary payments, inadequate or poorly structured incentive systems, and related problems such as systemic corruption. Other characteristics like poorly developed primary and secondary care resulting in overburdened tertiary level facilities are also common to these regions which result in specific distortions in care seeking and provision. For example, large numbers of pregnant/labouring women present at higher facilities with no prior contact and with complications, which resemble emergency care environments. The interventions we reviewed speak to these health system challenges that impact the provision of maternity care.<br><br>**Accountability:** There also may be significant differences in adherence to protocols and standards of practice on the whole, and how providers are held accountable for clinical care (and conversely malpractice) through institutional and legal processes.<br><br>Additionally, we were aware of a scoping review on organizational culture in maternal care that was underway which included all regions (https://osf.io/9cmuh). We wanted to avoid overlap or duplication of findings.
2) RMC is not merely the absence of D&A or mistreatment and therefore these terms cannot be used interchangeably. This study seems to explore the association of organizational challenges with the incidence of D&A, so this should be stated.<br><br>RMC is not the absence of D&A/mistreatment, so these terms should not necessarily be used interchangeably.<br><br>This paper seems to explore organizational factors that could be hypothesized to drive D&A...not RMC.	We agree that the hypothesised relationship is with mistreatment/D&A and thank you for noting this. We have revised the language and use RMC more selectively. Changes are indicated in track.
3) The strength of the evidence and the associations derived from the narrative review of qualitative and intervention studies should be discussed. Overall, it appears that this review provides a strong basis for hypothesis generation and calls for further research to explore the associations and their strength between the organizational issues (independent variable) and their effect on D&A (dependent variable)	While a critical appraisal of studies is not a requirement for scoping reviews and was not conducted here (to assess the strength of individual studies), we agree on the importance of raising hypotheses and pointing to further research to test these relationships and have included this in the discussion. Line 989-996.
4) There are a number of language issues that could be examined and addressed, such as	We agree that our use of organisational vs. institutional is confusing. We have modified the

whether there is a significant distinction between “organizational” and “institutional” and whether “irrational” and “rational” are commonly defined and understood as they are used in this paper. Professional copyediting would strengthen the writing.<br><br>What is the distinction? Are their organizational issues at another level? Organization = institution<br><br>Suggest another word choice, e.g., incoherent or unreasonable.<br><br>Suggest, “unreasonable” or “illogical” as “irrational” has the connotation of cognitively or emotionally impaired.<br><br>Same issue<br><br>This does not reflect common usage/definitions for this term.	language in several places to replace institutional with facility-level to improve clarity.<br><br>Rationality is a term widely used in economics and organisational theory. We use the concept of rationalisation of work as it is applied in the field of economics, referring to organisation or reorganisation of work for greater operating efficiency.<br><br>We refer to rational obstetric practice within the WHO framework of rational use of medicines which requires that “patients receive medications appropriate to their clinical needs, in doses that meet their own individual requirements, for an adequate period of time, and at the lowest cost to them and their community.”
The research emanating from the Heshima (PopCouncil) and Staha (AMDD) research projects identified complex and numerous drivers that should be mentioned.<br><br>Heshima project included study interventions aimed at caring for the carer, with mindfulness, self-care, etc.<br><br>https://bmcwomenshealth.biomedcentral.com /articles/10.1186/s12905-017-0425-8<br><br>https://www.popcouncil.org/research /respectful-maternity-care-resource-package<br><br>https://www.popcouncil.org/uploads /pdfs/2015RH_RMC_CaringForCarersBrief.pdf	Thank you. We have reviewed related papers and referred to the Heshima project. Kate Ramsey’s work which we referenced in the discussion, draws on the the Staha research.
An extensive body of literature that merits mention here is the nursing literature on caring.<br><br>Again, there is a entire body of nursing literature on caring and compassion.<br><br>Generally, compassionate care implies that the directionality of the compassion is from the carer to the person receiving care. If this is an alternative definition, it should be further described.<br><br>“Care for the carer” is more common, and was a focus of the interventions in the Heshima project specifically focused on addressing D&A and promoting RMC.	The majority of studies referenced in this paragraph are focussed on nurses. We have also included in the following: “The concepts of caring and compassion in relation to these working conditions have been examined in-depth in the nursing literature”, and added references. Line 45-46<br><br>Heshima project has been referenced in lines 54 and 944-947.
Surely, staffing volume is critical and deserves a specific mention. The workforce shortage (lack	Workforce shortages have been added, reflected in line 39-40.

of supply) and restricted staffing (economic motives) also occur in nursing and midwifery in HICs.	
I disagree with the dichotomization that is presented between HIC and LMIC with respect to power hierarchies in maternal newborn health care. Having worked for the national midwifery professional association in the US where I oversaw professional issues to provide technical and policy support to midwives across all 50 states, the phenomena described in this paragraph also apply in HIC settings.<br><br>Like D&A itself, the drivers of D&A are universal, while their manifestations and perhaps magnitude many vary contextually.<br><br>Still, it is a valid question to explore how they manifest in LMIC.<br><br><br>This presents a false dichotomy between HIC and LMIC. These factors apply also in HIC where nurses and midwives are nonetheless lower in the power hierarchy than physicians who are often in positions of legal ownership or executive/clinical leadership of the practices.	We are not claiming that shortages don't exist in HICs, with negative effects on providers and care. We are arguing that shortages take up a large focus of LMIC health system challenges and that other facility level-organisational factors have received relatively less attention.<br><br><br><br><br><br><br><br><br><br><br>Again, we are not claiming that these professional issues don't exist in HICs. In fact, we acknowledge in the previous paragraph that these issues are well established in HICs and better explored. Still, we have modified the following sentence to clarify:<br>"How work is divided, supervised, and rewarded are important factors for provider motivation and performance across all regions " Line 57-58
Should be cited as a reference:<br>WHO: Midwives Voices, Midwives Realities Findings from a global consultation on providing quality midwifery care.20 January 2016<br>| Global report	Thank you for this useful reference. We have included it. Line 897-898.
This framing is inherently hierarchical. In many settings, cadres function as independent collaborators in teams with flattened hierarchies, rather than in vertical structures where some supervise or monitor others.<br><br>The patient safety literature elevates flattened hierarchies as the safest cultures, and the healthcare domain adopted them from aviation safety.<br><br>Only in the literature on healthcare provision LMIC is there such an emphasis on supervision, rather than effective teamwork and workplace safety, which like QOC or health system research can have "hardware and a software" components.	We see supervision and monitoring as neutral terms in themselves and make the distinction between negative vs. supportive supervision later in the paper. We agree that the movement to less hierarchical structures in healthcare has been slower in LMICs but feel that this is why the terminology is still relevant for an exploration of organisational problems in these contexts.
Flowchart suggests papers were excluded if they did not focus on maternal health.	The non-intervention studies included to identify organisational problems were all maternal health. The intervention studies included to

Why are these listed as both reasons for exclusion and inclusion?	identify possible solutions were across disciplines.<br><br>Additional notation has been added to the flowchart to clarify.
From what range of disciplines, and what is rationale/evidence for generalizability?	The majority of intervention studies sampled multiple departments and looked at the facility as a whole. Nine of the thirteen studies included maternity/OBGYN departments which supports our ability to apply lessons for maternity care.<br>stating
What is the purported association between these issues and D&A/mistreatment, and how is this conceptualized, explored, substantiated in the methodology of this paper?<br><br>In some cases literature that demonstrates the association is cited but not in all cases. It would be stronger to reference studies that link the effects of workplace conditions to behaviors constituting D&A for each category described.	Given that scoping reviews are exploratory and specifically serve to map/organise literature, we do not feel hypotheses on expected associations are required to be introduced in the methodology and carried through the paper.<br><br>This is an under-explored area in LMICs with respect to D&A/mistreatment. We feel it is important to also include effects on providers that could relate to D&A/mistreatment, given that wider literature suggests that motivation, job satisfaction etc. have a bearing on how care is provided. As you suggested previously, it points to important areas for future research specifically designed to explore the link between organisational issues and mistreatment that these studies did not set out to do so.<br><br>We added these reflections on the strength of the evidence in the discussion. Line 989-996.
This is backwards: poor care is attributed to low staff morale, etc.	These papers reflected provider perspectives and reported provider views. To clarify, we have modified the sentence to read "The lack of rest with heavy workloads was noted to lower staff motivation and morale, productivity and concentration, which was linked with poorer or sub-optimal care". Line 238-239.
This itself is a manifestation of D&A/mistreatment, whereas in some instances the driver is only hypothetically linked to the behavior/manifestation.<br><br>Intentionally delaying care (line 258) should be included in this category.	That example has been moved to the paragraph on direct mistreatment. Change reflected in line 250.
Here, grammar and syntax need attention.	Sentence changed to "and were even perceived to be "working with anger and hatred""<br>Line 252-253
That sounds perfectly natural. Not smiling when tired and hungry can not be equated to disrespect and abuse. It is not reasonable to expect frontline workers to smile all the time but they can still behave kindly and respectfully. Perhaps there is another illustrative quote for this section.	We agree, this quote has been removed.

To maintain a consistent subject-object syntax in this sentence, this should read, "leaving clients unattended and untreated"	Recommended change made and reflected in line 264.
This grammar and syntax needs to be rewritten for improved comprehension.	Changes reflected in line 264-266.
And it is well documented in HIC as well. As stated above, the issues are universal across countries representing the full range of income levels, while the manifestations and their magnitude MAY vary. All of the examples listed here apply in HIC as well.	This paragraph is in the findings section and refers to the included LMIC studies.
Suggest rewriting as the wording is awkward and subjective.	Now reads "the more stable cadre with significant expertise" line 325.
Suggest making explicit that this is a manifestation of lack of privacy, a manifestation of mistreatment.	Teaching hospitals may not view this as lack of privacy, therefore for the findings section, we restrict this to how it was reported.
Suggest different word choice (colloquialism)	Now reads "securing care from doctors for obstetrics complications" Line 342.
Midwives ARE clinicians. This must be an artifact of translation from Turkish, but it is unacceptable in English to suggest that midwives are not clinicians. It perpetuates the lack of professional autonomy described in this section.	The quote conveys a crucial point about midwives subordination in multi-cadre teams and is revealing. We do not know for certain if the choice of the word is a translation error or a reflection of context specific power inequalities.
Looks like this should be removed.	"22" removed
This is an example of macro policy being inconsistent with national professional education, regulation, and association. As such it is separate from facility organizational issues and this should perhaps be noted. The suboptimal professional issues are pervasive and go beyond facility level.	We agree. Added "reflecting inconsistencies between policy and professional education that impact care provision". Line 416-417
It is questionable whether training and supervision should be lumped together.<br><br>This particular point seems to be more aligned with the guidance on clinical protocols described in the next paragraph.	This example was kept here because it involved the supervision of interns and therefore involves their training. We do appreciate your point and have moved it to the section on guidance on protocols.
Supervision is listed in multiple sections	The quality of supervision in LMIC contexts has implications for care provision beyond staff capacity and morale, and cannot as such be limited to one section.
Why "even in"?	Deleted.
Given that routine episiotomy is not evidence-based and should be considered a harmful practice, the fact that it is described so often raises the specter of poor provision of care.	Thank you for noting this. Though we do observe that episiotomy is mentioned across studies (and share concerns on its routine use), the papers themselves did not report that episiotomy was used routinely.
This paragraph seems to prevent a summarization of the results overall, and I	This paragraph was a summary of the first part of the analysis of observational/non-intervention

question why it appears here, rather than in the Discussion section of the paper.	studies before the intervention study results are reported.
Rather than saying “of relevance” it would be preferable to state “that have demonstrated effectiveness in improving RMC (or reducing D&A, more accurately) as well as some that have not been shown to be effective.”	These were not interventions designed to reduce D&A. We are highlighting aspects of the interventions that could be of importance when designing RMC interventions. We do not feel this wording should be changed.
Is there evidence to substantiate the link between job satisfaction and RMC or lack of D&A?<br><br>These assumptions and hypotheses should be made explicit in the Introduction and Methods sections of the paper and evidence should be presented with indication of the strength of such evidence.	Responding to your earlier suggestion, we have highlighted this as areas for future research.<br><br>Responded above to point (3)
Defined as...	“People orientation” is not defined within this paper and therefore will not be expanded on there. But Hee Jeon et. al. refer to Jeong-Eon et al.’s (2015) work which used a 7-item list including listening to employees, being trusted, making fair and balanced decisions, keeping the interests of employees in mind.
Yes, theoretically, but is there evidence that greater RMC is evidenced in facilities with a greater patient safety culture? If not, a statement like, “Further research should test this association” would be recommendable.	We have included such wording in the discussion section.
Suggest focusing on “institutionalization” of positive changes, especially since the focus is on institutional or organizational issues.	Changed to institutionalised.
Grammar and syntax.	Changed to “we also don’t know how long such reforms can survive if resource constraints and skewed workloads remain unaddressed for long periods.” Line 952-954.
Quality of scientific writing. Numerous colloquialisms.	Changed to “the review also raises questions on the structures and processes of power”.<br><br>“bosses’” replaced with “management’”<br><br>“questions on how far one can go” replaced with “the extent of organisational change” “
“emotional”?	Ramsey uses Riley and Weiss’ definition of emotion work, meaning “effort required to manage personal or others’ emotions in the workplace, such as service users and colleagues”
Narrative review is the lowest level of evidence in the hierarchy of quality of evidence.	We’ve made clear through the language and reporting standards of this paper (following established methodology, adhering to the PRISMA flow diagram for scoping reviews,

	providing a detailed search strategy etc.), this is a scoping review and not a narrative review. While systematic reviews are, of course, more robust methodologically, scoping reviews are particularly applicable to broad areas of exploration. Therefore, we do not feel our choice of review is a limitation.
This seems to be overreach and I would suggest focusing on hypothesis generation and formative research to substantiate the need for hypothesis-testing studies.	Now reads: "Second, given the strong emphasis on resource shortages as a primary barrier to poor care in public institutions, the review raises questions on its relative influence, pointing areas of organisational management that require attention when designing RMC interventions." Line 1025-1028.
The aim of the study was to explore organizational factors that may be associated with RMC (or more accurately D&A/mistreatment). This would be reflected in the summary statement.	These were not studies on mistreatment and therefore we are conscious of wording the objective as exploring facility-level organisational factors that impact provider behaviour and care.
"tackling"?	Changed to "addressing". Line 1027
**Reviewer 2**	**Author responses**
I think when we are researching issues such as this, we must recognise that there are always going to be dominant discourses, it's hard to explain why something happens -and it's so easy to blame being for example being overstaffed – and we have to develop research methods / approaches/ questions that go beyond the obvious.	We agree that drivers of mistreatment/D&A are many and interact in complex ways. We have added a sentence in the discussion to reflect this. Line 1004-1008.
In a former life – not as an academic but a government official – I found when visiting facilities that everyone said that they were overworked – but there was a huge variation of workloads. Some maternity wards were totally overloaded – some were overloaded some of the time – and some were not. Similarly, I found variation in the care that was given by staff – with some being nicer to patients than others – despite the challenging circumstances. I have always been frustrated that our methods and approaches/ reporting of work in this to issue often don't give space for individual variation. This not to say there are not structural issues – but I think there are also individual variations – and we need to think about how to support good providers!	Thank you for raising this point. We have added to the discussion. Please see lines 998-1004.
In the background (line 9/10) the author states that "we have significant evidence that women, especially those who are disadvantaged and marginalised, experience forms of mistreatment". In the results reported from the review there did not seem to be information reported about why organisational factors might lead to some women being treated worse than others – explanations were just given why overall treatment might be poor quality. Is this	We agree that this is point that needs further attention. We revisited our reviewed papers and differences in how women were treated along socio-economic lines and other axes of identity did not emerge strongly across papers. We think this may be because these were largely qualitative studies using provider interviews that would have more easily yielded answers on challenging working environments (compared to women's report of experience, user surveys and observation).

because it was not explored in the literature? I think it would be worth reflecting on.	We have raised questions on this in the discussion for future research. Line 998-1004.
I had similar questions about ‘structural gender inequality’ and ‘women’s low status in the community’. Did any of the articles address these issues?	As the driver in focus for our review was organisational problems in facilities, we did not systematically extract findings related to structural gender inequality. We have removed the sentence in the introduction in case it implies that we will be exploring this issue further in the review.
Another question that I had – and maybe worth at least addressing in the discussion is around the specifics of disrespect and abuse in maternity services. I found myself thinking when I read the article – that workload/ poor supervision/ organisational dynamics – don’t seem that particular to maternity services – but there do seem to be some specific problems that exist in maternity services.	Thank you for raising this point. We believe that organisational issues interact with other drivers of disrespect and abuse related to structural gender inequality for D&A to manifest this way in maternity care specifically. We expect that attention to organisational challenges combined with community/societal level interventions (or interventions targeted at providers) to challenge context specific gender norms and biases will ultimately be more effective to address D&A.
So, in conclusion – this is a great article – but I found myself wanting a better link between the intro and discussion – and either extraction of data related to social inequalities – or recognition that this is not being addressed.	We have added additional wording in the discussion to reflect our intention to isolate organisational factors and that further research should explore how different drivers interact. Line 1004-1008.
